# VIVALDI Cohort Profile: Using linked, routinely collected data and longitudinal blood sampling to characterise COVID-19 infections, vaccinations, and related outcomes in care home staff and residents in England

**DOI:** 10.12688/wellcomeopenres.20278.2

**Published:** 2024-08-19

**Authors:** Maria Krutikov, David Bone, Oliver Stirrup, Rachel Bruton, Borscha Azmi, Chris Fuller, May Lau, Juliet Low, Shivika Rastogi, Igor Monakhov, Gokhan Tut, Douglas Fink, Paul Moss, Andrew Hayward, Andrew Copas, Laura Shallcross

**Affiliations:** 1 Institute of Health Informatics, University College London, London, England, NW1 2DA, UK; 2 Institute of Immunology and Immunotherapy, University of Birmingham, Birmingham, England, B15 2TT, UK; 3 Institute for Global Health, University College London, London, England, WC1N 1EH, UK; 4 UK Health Security Agency, London, SW1P 3JR, UK; 5 Division of Infection & Immunity, University College London, London, England, WC1E 6JF, UK; 6 Institute of Epidemiology & Health Care, University College London, London, England, WC1E 7HB, UK; 7 Health Data Research UK, London, England, NW1 2BE, UK; 8 University College Hospitals London NHS Trust, NIHR Biomedical Research Centre, London, NW1 2PG, UK

**Keywords:** COVID-19, care homes, older adults, infection, data linkage

## Abstract

VIVALDI (ISRCTN14447421) is a government-funded longitudinal open observational cohort study of staff and residents in care homes for older people in England. The study aimed to describe epidemiology (including seroprevalence) and immune responses to COVID-19 in a subset of care homes, in the context of extremely high mortality in this setting, in the first 12-18 months of the pandemic. Data linkage to routine health data was undertaken for all staff and residents and a subset of individuals who consented to sequential blood sampling to investigate SARS-CoV-2 immunity. This paper aims to describe the samples stored within the VIVALDI biobank and associated linked data, available for use by researchers.

Over 70,000 individuals from 346 care homes were included in the data linkage cohort (1 ^st^ March 2020–31 ^st^ March 2023). 4971 samples from 2264 individuals (1415 staff, 827 residents) collected between 29 ^th^ October 2020 and 10 ^th^ March 2023 are stored. Amongst these samples, there was a maximum of seven per participant however, 217 (26.2%) residents and 551 (38.9%) staff participated in one round only.

Key study findings include high COVID-19 seroprevalence among surviving residents, exceeding rates in community-dwelling peers. COVID-19 vaccinations generated robust immune responses in staff and residents which waned, supporting the need for booster vaccination, particularly in response to new variants. Prior infection significantly improved vaccine-induced immune responses, however protection from infection declined following Omicron variant emergence.

This is a unique cohort of pre- and post-infection samples linked to data on COVID-19 infections, vaccinations, and outcomes. The cohort spans host immune response evolution to infection and vaccination in this rarely sampled population of frail older care home residents who are especially vulnerable to infection and severe outcomes. These samples can be used to investigate biological mechanisms behind disparate infection responses in older people and make a valuable contribution to research into ageing.

## Introduction

The VIVALDI study (ISRCTN 14447421) is one of the largest studies of Coronavirus disease 2019 (COVID-19) in care homes for older people globally. The study was rapidly established early in the pandemic ^1^ when over one-third of COVID-19 associated deaths in England had occurred in care home residents, even though residents account for less than 1% of the general population ^2^. Whereas there is well-established surveillance for infections in the community, infection monitoring in social care is under-developed because of limited clinical and data infrastructure ^3, 4^. During the pandemic this was compounded by delays in establishing *de novo* polymerase chain reaction (PCR) testing capacity. The study aimed to address this gap in COVID-19 surveillance and research by establishing a serological survey and cohort study to measure seroprevalence, immunity, and responses to vaccination amongst male and female care home staff and residents in England. As most of this population are female, it was expected that females would predominate in the sampled cohort.

VIVALDI was a core COVID-19 surveillance and immunity study, funded by the UK Government Department of Health & Social Care and the UK Health Security Agency to inform the public health response in care homes. Study investigators worked closely with national policymakers throughout the study to translate research findings into policy and practice, with the aim to reduce severe acute respiratory syndrome coronavirus 2 (SARS-CoV-2) infection transmission within care homes and the general population ^3^. In the cohort study, data linkage was performed to routinely collected health data from everyone in participating care homes. The survey consisted of additional blood sampling in a subset of this population for immunological testing. Here we describe the cohort of residual blood samples that are available for further research.

## Methods

### Consent

Participants or their consultees have provided written informed consent for storage of blood samples for further research (detailed description below). In accordance with this approval, samples will be stored in UK Biocentre. All samples belong to participants who have consented to their further use by researchers outside of the VIVALDI team (n=2264, 1415 staff, 827 residents, 22 unknown) using two different consent questions, either for research relating to COVID-19 only (n=1,290, 389 residents, 887 staff, 14 unknown) or to wider research around ageing (n=974, 438 residents, 528 staff, 8 unknown).

### Ethics approval

The study has been approved by the South Central – Hampshire B Research Ethics Committee (20/SC/0238) on 29 ^th^ May 2020.

The legal basis for accessing data without consent was initially under the Control of Patient Information Regulations 2002 (notice in place between March 2020 and June 2022). The study obtained section 251 support from the Health Research Authority’s Confidentiality Advisory Group (CAG) (ref 21/CAG/0156) in March 2022.

### Who is in the cohort?

**Figure 1.**
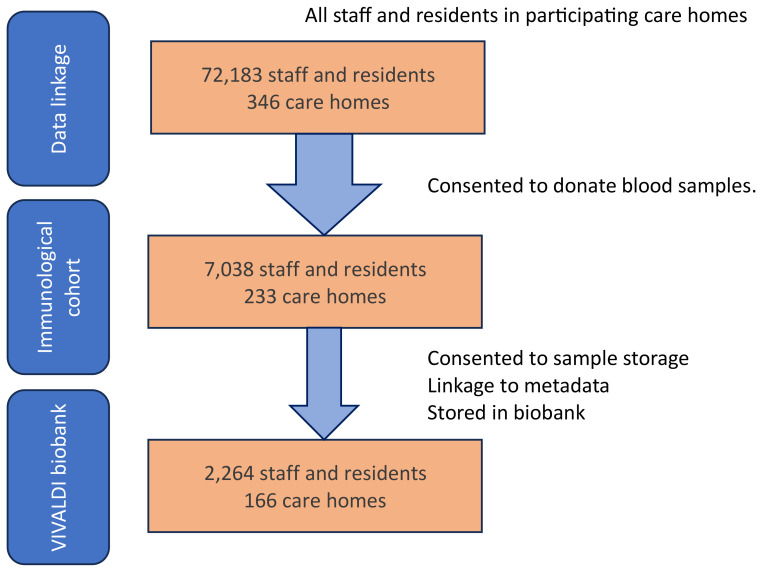
Cohort flow diagram. Three study cohorts are demonstrated; data linkage consisting of 72,183 participants from 346 care homes, immunological cohort with 7,038 staff and residents from 233 care homes, and the VIVALDI biobank cohort of 2,264 individuals from 166 care homes described in the cohort profile.

To provide context, the overall VIVALDI data linkage cohort is described below before focussing on the immunological cohort of stored samples. The cohorts are illustrated in Figure 1.

***Data linkage cohort.*** VIVALDI is an open cohort comprising all residents and staff from 346 participating care homes for older people distributed across England. Care homes are owned by a range of large and small care providers and include both for-profit and not-for-profit homes providing a mix of residential, nursing, and specialist dementia care. Recruitment of care homes was opportunistic and new care homes were able to join and leave at any point between 29 ^th^ May 2020 and 31 ^st^ March 2023. The study consists of data linkage to routine surveillance data from the entire cohort and a smaller seroprevalence cohort, which is the focus of this cohort profile, described in more detail in “what has been measured?”. All individuals with SARS-CoV-2 tests that were linked to a participating care home identifier (Care Quality Commission ID, CQC-ID) were included. As regular asymptomatic PCR testing was routinely performed in all care homes from approximately September 2020 onwards (at least weekly in staff and monthly in residents) ^4^, dates of PCR testing could be used to infer dates of care home entry and exit in staff and residents. The precision of estimates of the exact number of participants are limited by data entry errors or incomplete data, however 72,183 participants could be linked to a valid NHS number.

***Immunological cohort.*** A subset of 7,038 individuals from 233 care homes gave written informed consent to participate in a population-based seroprevalence survey spanning between 11 ^th^ June 2020 and 31 ^st^ March 2023. Recruitment was opportunistic and sampling frames to ensure representation by sex or age were not applied. Sampling was organised in rounds but had to account for phlebotomy capacity and care homes' closures due to outbreaks. Participants donated sequential blood samples over the study period. As this was an open cohort, participants were able to enter and exit at any point and all staff and residents at homes with blood sampling visits were eligible to take part. Linkage to routine data, available from the wider VIVALDI cohort, was undertaken for participants who supplied a valid National Health Service (NHS) number as this enabled identification of participants within the pseudonymised data, detailed description in “what has been measured?” section.

Consent was led by senior care staff at each care home with general oversight from a provider-specific project manager. This approach was taken primarily because it was not possible for external researchers to visit care homes during the pandemic, and there was an urgent need for data on seroprevalence to inform public health action. Care homes were also geographically dispersed across England and there were strict restrictions on population movement at the time of recruitment. Study information was distributed by care providers to all next of kin and followed up with a phone call to check understanding and discuss specific queries relating to the study. For individuals lacking capacity to consent, personal or nominated consultees were identified from next of kin or care home staff, respectively.

Samples were tested for the presence of infection and vaccine derived IgG antibodies against SARS-CoV-2 (anti-nucleocapsid and anti-spike respectively) and more in-depth components of the immune response to infection. Until April 2022, all samples were tested for the presence of anti-nucleocapsid IgG antibodies using the Abbott ARCHITECT i-system (Abbott, Maidenhead, UK) immunoassay. Samples collected beyond this date were tested for anti-spike and anti-nucleocapsid IgG antibodies using the Roche Elecsys immunoassay (Roche Diagnostics International Ltd, Rotkreuz, Switzerland) following emergence of data showing greater sensitivity and lower susceptibility to waning using this assay compared with Abbott ^5– 7^. Collaborators at the University of Birmingham and the Francis Crick Institute have undertaken additional testing of a subset of samples including quantitative humoral and cellular responses to different SARS-CoV-2 variants and other seasonal coronaviruses.

***Stored samples (VIVALDI biobank).*** A total of 4,971 residual serum samples that could be linked to wider surveillance data from 2,264 individuals who consented to storage of samples for future research are held in a registered biobank (UK Biocentre, Milton Keynes): www.ukbiocentre.com. As storage was carried out according to availability of data and samples, sex of participants was not considered when selecting samples. These samples were collected between 29 ^th^ October 2020 and 10 ^th^ March 2023.

Of these, 827 residents (36.5%) donated 2,118 samples, 1,415 staff (62.5%) donated 2,817 samples, and 22 individuals with unknown subject type (1.0%) contributed 36 samples. The median age was 89 (IQR 82-94) years for residents and was 52 (39–59) years for staff. Overall, 589/827, 71.2% of residents and 1,241/1,415, 87.7% staff were female. A total of 332 deaths (228, 68.7% female) occurred amongst participants over the study period and the median time between the final sample date and death was 201 days (86-365).

There were a total of 1,369/2,264 (60.5%) participants in whom SARS-CoV-2 infection was recorded over the study period using either Polymerase Chain Reaction (PCR) or Lateral Flow Device (LFD) tests. Of these, 934 (235 residents, 692 staff, 7 unknown) were sampled prior to infection, of which 786 were female (84.2%) and 608 were sampled following the infection (265 residents, 336 staff, 7 unknown) of which 502 (82.6%) were female (the remainder were male). Of 2,264 participants, 1,965 (86.8%) were sampled following vaccination (1,606, 81.7% female). Most received two vaccine doses over the period that samples were collected (1,034/2,264, 45.7%) and a maximum of six doses (3/2,264, 0.1%).

Participants were sampled from all seven regions of England, most frequently from the South-West (524/2,264, 23.1%) and least from London (101/2,264, 4.4%). A greater proportion worked or lived in care homes owned by not-for-profit compared with for-profit providers (1,061 *vs.* 965) and 227 were from independent care homes.

### How often have participants been followed up?

Amongst the stored samples, participants donated between one and seven blood samples over the study period. The median time between samples was 91 days (IQR 61-119), comparable between staff and residents (91 days). Table 1 illustrates the number of samples per participant, 1,484 donated more than one sample and 819 donated at least three samples.

**Table 1.** Total samples stored per participant. Total number of samples stored from each participant are shown for the whole cohort and by subject type and sex. Up to seven samples have been stored per participant. Most participants have donated up to three samples.

Total samples	Resident		Staff		Unknown		Total	
	Overall	Female, n (%)	Overall	Female, n (%)	Overall	Female, n (%)	
1	217	154 (71.0)	551	484 (87.8)	12	9 (75.0)	780
2	174	123 (70.7)	485	428 (88.2)	6	5 (83.3)	665
3	279	202 (72.4)	275	235 (85.5)	4	1 (25.0)	558
4	83	58 (69.9)	57	53 (93.0)	0	0 (0.0)	140
5	61	44 (72.1)	40	35 (88.0)	0	0 (0.0)	101
6	12	7 (58.3)	7	6 (85.7)	0	0 (0.0)	19
7	1	1 (100)	0	0 (0.0)	0	0 (0.0)	1
Total	827	589 (71.2)	1415	1245 (88.0)	22	15 (68.2)	2264

**Figure 2.**
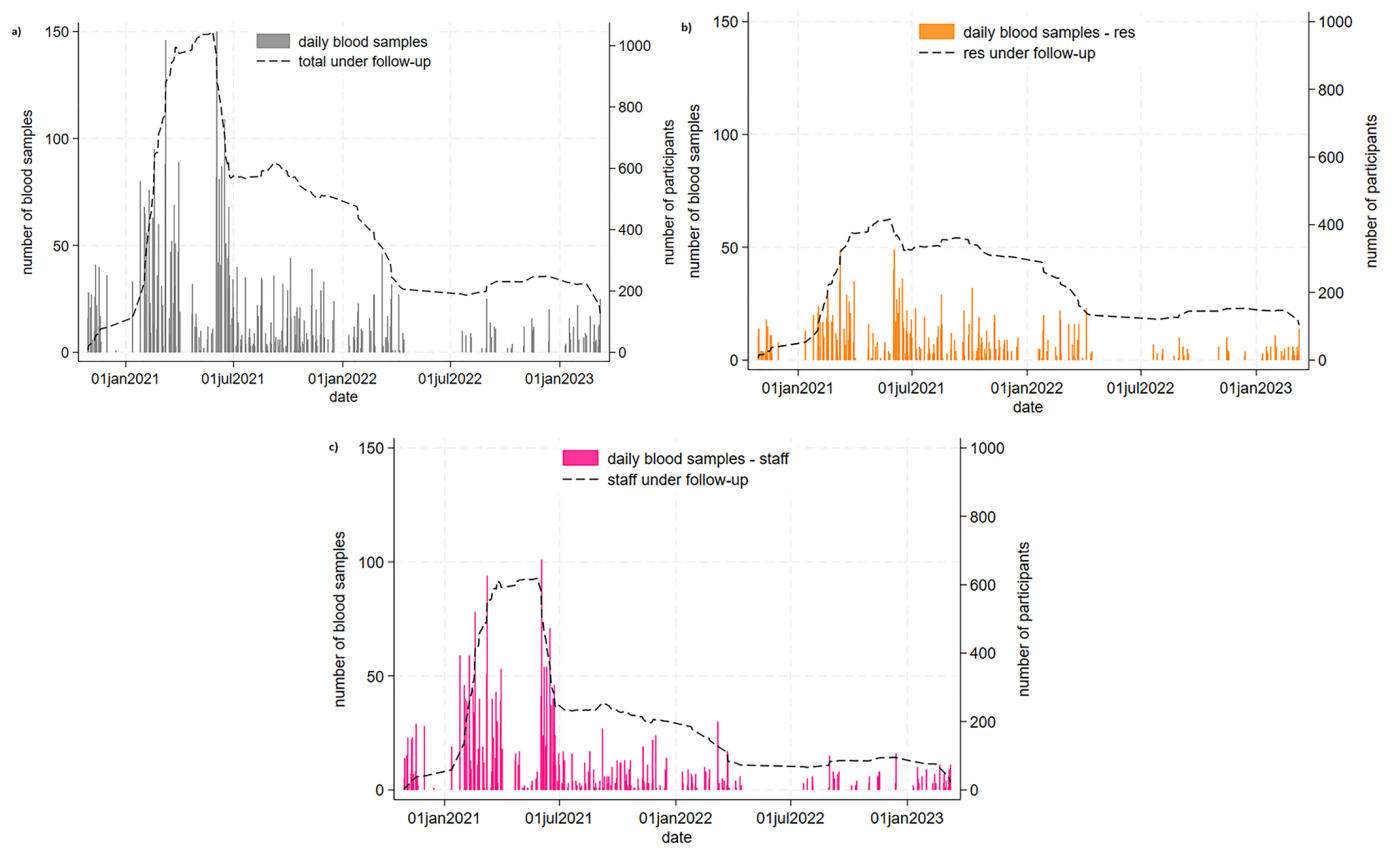
VIVALDI biobank cohort recruitment and sampling (October 2020 – March 2023). **a**) Overall, **b**) residents and **c**) staff. Plots show number of blood samples collected per day and number under follow-up. Number under follow-up are estimated based on date of first and final blood sample collection.

Most care homes joined the study within the first year of recruitment (June 2020 to May 2021) and most blood samples were donated over this period, Figure 2a–c. From June 2021 onwards, there was a rapid decline in participants under follow-up, greater amongst staff than residents.

### What has been measured?

Within the data linkage cohort, linkage to routine surveillance data has been conducted using unique pseudo-identifiers based on individual NHS numbers for individuals joining the cohort between 1 ^st^ March 2020 to 31 ^st^ March 2023, with linkage to new events for existing participants until 31 ^st^ March 2024. Results from immunological testing of blood samples have been linked to the data linkage cohort (pseudonymised study dataset). From 1 ^st^ March 2021 onwards 69.6% (6601/9486) of all blood samples received could be linked to a valid NHS number. Individual data linkage rates can only be estimated after this date as prior to this unlinked samples were excluded from the dataset. Only linked samples were included in the VIVALDI biobank cohort.

Linked data includes results of COVID-19 testing performed asymptomatically, symptomatically and during outbreaks. Universal asymptomatic screening in care homes commenced in June 2020 and was performed regularly using both PCR and LFDs as part of the national COVID-19 surveillance programme ^8^. This continued until the end of March 2022 in residents and August 2022 in staff, at which point testing was conducted for symptomatic individuals and to support outbreak investigation and management. Vaccination records includes dates and types of vaccines administered and were available from NHS Digital’s National Immunisation Management System (NIMS) ^9^. Dates of hospital admission and discharge and diagnostic ICD-10 codes were available from Hospital Episode Statistics (HES) ^10^ and dates and diagnostic codes for any deaths were recorded by Office for National Statistics (ONS). Demographic data including age, sex, care home, and care home role were available from COVID-19 testing records, which were entered by requestor during the test registration process (self or by care home staff member). Data describing comorbidities were retrieved from hospital admission records (HES) therefore are only available for participants with admissions during the study period and accuracy is subject to quality of recording at the time of discharge. Metadata that will be made available to researchers are outlined in Table 2.

**Table 2.** Metadata available to researchers. Metadata linked to samples that can be made available to researchers. Data items include demographics, COVID-19 test results, COVID-19 vaccinations, hospitalisations related to COVID-19, comorbidities, and death related to COVID-19.

Data item	Source
Demographics: age band (on entry), sex, care home role, region	Pillar 2
COVID-19 test results: any positive PCR or LFD tests prior to sample date	Pillar 2, Pillar 1
COVID-19 vaccinations: number of vaccines before sample date, vaccine types	NIMS
Hospitalisations: any COVID-19 admission before sample	HES
Comorbidities: presence of diabetes, cancer, dementia	HES
Death: COVID-19 death following sample	ONS

NIMS: National Immunisations Management System; HES: Hospital Episode Statistics; ONS: Office for National Statistics; COVID-19: Coronavirus disease 2019; PCR: polymerase chain reaction; LFD: Lateral Flow Device. Pillar 1 – UKHSA-led national COVID-19 testing programme to detect infections during care home outbreaks or during hospital admission. Pillar 2 – DHSC-led national COVID-19 testing programme to detect asymptomatic and symptomatic infections in the community as well as routine asymptomatic screening amongst care home staff and residents ^12^.

Linkage was also undertaken using CQC-IDs from participating care homes to the Capacity Tracker dataset ^11^. This was developed by North of England Care System Support (NECS) in partnership with NHS England and was used by the Department of Health & Social Care and Local Authorities to monitor care home capacity, staffing, training, and supplies.

Between April and November 2020, an opportunistic sample of 134 care homes (belonging to two for-profit and two not-for profit providers) participated in a one-off survey of the care home built environment, which was designed by public health, infectious diseases, and building science experts. Questionnaire responses were linked to by the CQC-ID to the study dataset.

## Dataset validation

To compare the sampled participants to the wider cohort within the care homes, we considered individuals who were present within the three months preceding the date of first blood sample taken at each participating care home and compared individuals with and without stored samples. Only individuals who could be linked to pseudonymised surveillance data could be included in this analysis as it was not possible to identify unlinked individuals within the dataset. Overall, a median of 10.0% (IQR 5.4–17.5) of each care home was sampled, 6.4% (1.4–13.7) of residents and 13.1% (5.4–21.8) of staff. Demographics did not differ substantially between groups although the proportion of females was higher in the sampled compared with unsampled group (81.5% vs. 74.0%), Table 3.

**Table 3.** Demographics of sampled and unsampled individuals, overall and in residents and in staff. A lower proportion of residents and higher proportion of staff are represented in the sampled when compared with the unsampled population. A lower proportion of residents are female when compared with staff and the sampled staff have a greater female proportion when compared with unsampled staff. Age distributions are comparable between groups. A greater number of samples were obtained from staff than from residents up until April 2022, after which the proportion equalised within the sampled population with slight predominance of staff in the unsampled population over the same period. However smaller numbers were recruited over this period, reflecting a shift in the study’s focus to detailed investigation of immune responses in a sub-cohort from March 2022. As the national testing programme wound down over the final months of the study, the number of COVID-19 tests being conducted dropped rapidly making it more challenging to identify the unsampled care home population within routine datasets.

Demographics	Sampled (n=2,264)		Not sampled (n=16,525)	
	Resident	Staff	Resident	Staff
Total, n (%)	827 * (36.6%)	1,415 * (62.5%)	8,116 ^±^ (49.1%)	8,245 ^±^ (49.9%)
Female, n (%)	590 (71.2%)	1,241 (87.7%)	5,379 (66.3-%)	6,740 (81.7%)
Median age, years (IQR)	89 (82-94)	51 (39-59)	88 (82-93)	46 (33-57)
*Date of first sample 01/04/20 – 31/03/21*				
Total, n (%)	503 (60.8%)	999 (70.6%)	5779 (71.2%)	5369 (65.1%)
Female, n (%)	364	882	3821 (66.1%)	4463 (83.1%)
Median age, years (IQR)	90 (83-94)	52 (40-60)	88 (82-93)	45 (33-57)
*Date of first sample 01/04/21-31/03/22*				
Total, n (%)	251 (30.4%)	342 (24.2%)	2081 (25.6%)	2502 (30.3%)
Female, n (%)	180	302	1391 (66.8%)	1984 (79.3%)
Median age, years (IQR)	88 (80-93)	50 (38-59)	88 (81-93)	47 (33-57)
*Date of first sample 01/04/22 – 30/03/2023*				
Total, n (%)	73 (8.8%)	74 (5.2%)	256 (3.2%)	374 (4.5%)
Female, n (%)	45	57	167 (65.2%)	293 (78.3%)
Median age, years (IQR)	89 (84-92)	53 (39-59)	87 (80-92)	46 (35-56)

Participants with unknown subject type not shown *n=22 ±n=164

To consider the retention of participants within the cohort of stored sample, we divided the cohort into participants with one sample and those who remained under serological follow-up with more than one samples, Table 4. Overall, these groups were similar in demographic makeup (including sex and age) however there was a greater proportion of residents who remained under follow-up when compared with staff. This is likely to be partly explained by the high rates of staff turnover in care homes, as most staff that remained under follow up worked in not-for-profit homes (62.5%), which have been reported to have experienced lower staffing shortages than for-profit homes over the pandemic ^13^.

**Table 4.** Demographic composition of participants with one stored sample compared with more than one sample, overall and in residents and in staff. More residents remained under follow-up (more than one sample group) when compared with staff. A greater proportion of residents under follow-up were female compared with those donating one sample only however this pattern was reversed amongst staff. A greater proportion of participants from for-profit homes donated one sample only, whereas a greater proportion of participants from not-for-profit homes remained under follow-up. Primary samples were most frequently collected early on before April 2021, least frequently in the final year of the study (1 ^st^ April 2022 – 1 ^st^ April 2023). A larger proportion of participants who donated their first sample before April 2021 provided at least one further sample during the study (1061/1484, 71.5% vs 456/780, 58.5%), although participants entering in the final months of the study may have had fewer opportunities to do so.

Demographics	One sample only (n=780)			More than one sample (n=1,484)		
	Total	Residents	Staff	Total	Residents	Staff
Total, n (%)	780 ^§^ (34.4%)	217 (27.2%)	551 (70.6%)	1,484 ^±^ (65.5%)	610 (41.1%)	864 (58.2%)
Female, n (%)	651 (83.4%)	154 (71.0%)	484 (87.8%)	1,198 (80.7%)	435 (71.3%)	757 (87.6%)
Median age, years (IQR)	57 (42, 77)	90 (84-94)	50 (37-59)	63 (50, 87)	89 (82-94)	53 (41-60)
Date of first sample, n (%)						
01/04/20 – 31/03/21	456 (58.5%)	113 (52.1%)	338 (61.3%)	1061 (71.5%)	390 (63.9%)	661 (76.5%)
01/04/21-31/03/22*	260 (33.3%)	73 (33.6%)	183 (33.2%)	337 (22.7%)	178 (29.2%)	159 (18.4%)
01/04/22 – 30/03/2023*	64 (8.2%)	31 (14.3%)	30 (5.4%)	86 (5.8%)	42 (6.9%)	44 (5.1%)
Region, n (%)						
East Midlands	96 (12.3%)	30 (13.8%)	66 (12.0%)	198 (13.3%)	61 (10.0%)	136 (15.7%)
East of England	50 (6.4%)	10 (4.6%)	40 (7.3%)	104 (7.0%)	55 (9.0%)	49 (5.7%)
London	43 (5.5%)	22 (10.1%)	20 (3.6%)	58 (3.9%)	31 (5.1%)	27 (3.1%)
North East	99 (12.7%)	33 (15.2%)	66 (12.0%)	136 (9.2%)	75 (12.3%)	60 (6.9%)
North West	98 (12.6%)	23 (10.6%)	73 (13.2%)	125 (8.4%)	50 (8.2%)	75 (8.7%)
South East	144 (18.5%)	29 (13.4%)	112 (20.3%)	376 (25.3%)	151 (24.8%)	222 (25.7%)
South West	127 (16.3%)	31 (14.3%)	93 (16.9%)	397 (26.8%)	128 (21.0%)	264 (30.6%)
West Midlands	55 (7.1%)	19 (8.8%)	34 (6.2%)	52 (3.5%)	36 (5.9%)	16 (1.9%)
Yorkshire & the Humber	68 (8.7%)	20 (9.2%)	47 (8.5%)	38 (2.6%)	23 (3.8%)	15 (1.7%)
Ownership, n (%)						
For-profit	413 (53.9%)	137 (63.1%)	271 (49.2%)	552 (37.2%)	301 (49.3%)	249 (28.8%)
Not-for-profit	249 (31.9%)	30 (13.8%)	217 (39.4%)	812 (54.7%)	258 (42.3%)	546 (63.2%)
Independent	116 (14.9%)	48 (22.1%)	63 (11.4%)	111 (7.5%)	42 (6.9%)	69 (8.0%)

^±^10 with unknown subject type not shown ^§^12 with unknown subject type not shown

### Strengths and weaknesses

A key strength of this cohort is the representation of frail individuals from the upper extreme of the age spectrum as one-third reside in care homes, the majority of whom are older than 80 years. Although longitudinal cohorts describing ageing exist, none include care home residents. Most recruited 40-to-60 year olds at baseline who have not been followed up or have not yet aged beyond 80 years ^14– 16^. Care home residents are a highly comorbid population with a significant prevalence of frailty, dementia, physical dependency, and malnutrition ^17, 18^ and as demonstrated over the COVID-19 pandemic, they are particularly susceptible to severe infections ^2, 19, 20^. The biological mechanisms behind this disparity are still not fully understood therefore high-quality research focussed in this population could inform interventions to limit the negative consequences of infection. The inclusion of staff who worked in the same care homes over the sampling period also provides a useful comparator group of younger individuals. The proportion of the cohort that are female is consistent with data from the sector showing that 83% of workers and almost three-quarters of residents are female ^21, 22^, suggesting that this a broadly representative sample. This is important as it is well-recognised that there are distinct differences in pathophysiology of infection between males and females, which are likely to account for differences in infection rates and outcomes. This also suggests that study findings to date can be generalised to the overall care home population. This is a particularly valuable resource as care homes and women are frequently excluded from clinical studies due to the logistical challenges associated with recruiting and following up participants.

In addition to the composition of the cohort, linkage to surveillance data on infections, vaccinations, and outcomes over the entire study period is a significant strength. Sequential samples were collected over a period of high COVID-19 incidence with linkage to high-quality data on asymptomatic infections. This has maximised infection ascertainment and provides a unique opportunity to study temporal responses to infection and consider factors associated with severe outcomes in pre- and post-infection samples. As samples have also been collected before and after COVID-19 vaccination, this is a unique opportunity to mechanistically study immune responses to vaccination and vaccine effectiveness.

The cohort is limited by completeness of follow up as 34% of participants only donated one sample. For 204 of these participants (26%), this is due to logistic issues as 34 out of 166 care homes only contributed one round of samples to storage. In addition, high turnover of staff and residents, in part due to high resident mortality and significant staff movement over the pandemic may have accounted for loss to follow up. The average resident life expectancy in a care home is estimated to be 12–24 months ^23, 24^ and in 2021/2022, the average staff turnover in the care sector in England was 29% ^25^. The study also recruited temporary residents, frequently admitted from hospital while awaiting step-down care who may only spend a few weeks in a care home but are likely to have been recently exposed to infection. In addition, only individuals that could be linked to surveillance data have been included, mainly those who were able to supply their NHS number (enabling data linkage to pseudonymised data). Furthermore, data on comorbidities are only available from hospital admission records which may be incomplete and are missing for participants without recorded admissions. As ethnicity data from surveillance records was unreliable or missing, we elected to remove this from the metadata to limit bias.

## Patient and Public Involvement (PPIE)

The study design was reviewed by representatives of the National Care Forum, an organisation for not-for-profit care providers. Throughout the study we have regularly reviewed the acceptability of study plans with senior staff at participating care homes to ensure they are feasible and acceptable. In view of rapid study timelines, there was insufficient time to engage more widely before the study commenced however over the first year we formed two study-specific PPIE groups, consisting of care home staff and relatives of residents. These groups assisted in translating key study findings into lay summaries, which were distributed within participating care homes and published on the study website [ https://www.ucl.ac.uk/health-informatics/research/vivaldi/vivaldi-patient-and-public-engagement]; both received excellent feedback. We have visited five different care homes to discuss our study findings to date, progress, and future plans with staff and residents. During interviews with at least ten members of care home staff where we discussed their attitudes towards research and data sharing, many expressed wide support for the study. The VIVALDI symposium was held mid-way through the study and was attended by key stakeholders including academics, policymakers, and care home staff, where news about the study were shared and future plans discussed in an informal setting. These activities and collaborations were highly valued by all stakeholders and have been key to the study’s success.

## Key findings to date

VIVALDI study findings have directly informed the national COVID-19 pandemic response, most significantly they were among the first to describe vaccine effectiveness, mortality, reinfection risk, and the spread and impact of SARS-CoV-2 variants in care homes. A full list of >15 peer-reviewed study publications is available on the VIVALDI study website [ https://www.ucl.ac.uk/health-informatics/research/vivaldi/vivaldi-publications].

The immunological cohort has been used to describe seroprevalence and immunological responses to infection and vaccination. Analysis of anti-nucleocapsid antibody results from over 9,000 blood samples collected over the first 15 months of the pandemic, demonstrated that at least one-quarter of staff and one-third of residents had experienced SARS-CoV-2 infection over this period, which was greater than infection prevalence in age-matched peers living in private residences ^26^. As reliably measuring COVID-19 incidence in care homes had been challenging over the early months of the pandemic, these findings highlighted the increased risk of infection in care home residents, despite infection control measures. This risk is likely to be partly explained by staff and resident movement between community and secondary care settings, and close contact between staff and residents in the care home environment.

We have also examined immunological responses to infection and vaccination in a sub-set of participants ^27– 30^. We demonstrated comparable humoral and cellular responses amongst staff and residents following infection ^28^. In vaccine recipients, these responses developed more rapidly and were consistently larger amongst previously infected compared with infection naïve participants ^27, 29^. Although they were comparable in magnitude and rate of decline between staff and residents, there was some variation depending on vaccine type ^31^. Evaluation of responses to third dose vaccinations showed enhancement of antibody responses most significantly amongst residents and neutralisation of the emergent and rapidly dominant Omicron variant (from December 2021). However, linkage to infection data demonstrated antibody decline within 100 days of third vaccination and within 24 weeks of vaccination, breakthrough infections occurred in one-quarter of participants from 7 weeks onwards ^30^. Taken together, in view of high incidence of SARS-CoV-2 infection within care homes over the pandemic, residents are likely to exhibit enhanced immune responses to infection when compared with peers living in private residences, however there is still evidence of waning of responses over time. This work has provided important evidence for national revaccination campaigns that prioritise older adults and care home residents, especially since emergence of the Omicron variant.

## Data Availability

Researchers who would like to access samples and linked metadata can submit an expression of interest on the UCL VIVALDI study website [ https://www.ucl.ac.uk/health-informatics/research/vivaldi/vivaldi-serum-biobank]. In view of restrictions on data sharing, metadata will only be available for linked samples and cannot be shared for the wider data linkage cohort who have not consented to sampling. Requests will be reviewed by the study team, who will subsequently provide an estimated cost for samples. Ethical approvals must be in place for any further analyses of the samples and data. Any publications must acknowledge study participants and credit the VIVALDI study team.
