# Supplementary material for: VIVALDI Cohort Profile: Using linked, routinely collected data and longitudinal blood sampling to characterise COVID-19 infections, vaccinations, and related outcomes in care home staff and residents in England
Source: Wellcome Open Res. 2024 Aug 19;8:553. Originally published 2023 Dec 4. [Version 2] doi: 10.12688/wellcomeopenres.20278.2 (PMC11447437; doi:10.12688/wellcomeopenres.20278.2)
Supplement: Peer review history

# Reviewer report: Reviewer response for version 2

**DOI:** 10.21956/wellcomeopenres.25302.r100271

**Published:** 2024-10-22

**Authors:** Marc Ka-chun Chong

**Affiliations:** 1 The Chinese University of Hong Kong, Hong Kong, Hong Kong

In most of the existing cohorts of COVID-19 infected cases, the blood data are usually lacked - this project initiated a valuable cohort that linked longitudinal blood data, COVID-19 infection infection, vaccination records, and other related data in elderly. The dataset is highly useful for informing disease control strategies based on the seroprevalence data. Two comments:

 1. Any immune response data to specific variants (e.g JN1)?

 2. It seems the sampling time between samples is longer than I expected that short term response curve cannot be established.

Are sufficient details of methods and materials provided to allow replication by others?

Yes

Is the rationale for creating the dataset(s) clearly described?

Yes

Are the datasets clearly presented in a useable and accessible format?

Yes

Are the protocols appropriate and is the work technically sound?

Yes

Reviewer Expertise:

Infectious diease epidemiology

I confirm that I have read this submission and believe that I have an appropriate level of expertise to confirm that it is of an acceptable scientific standard.

# Reviewer report: Reviewer response for version 2

**DOI:** 10.21956/wellcomeopenres.25302.r94000

**Published:** 2024-10-02

**Authors:** Sebastian Maurer-Stroh

**Affiliations:** 1 Agency for Science, Technology and Research (A*STAR), Bioinformatics Institute, Matrix, Singapore; 2 National University of Singapore, Singapore, Singapore

The authors have sufficiently addressed all concerns.

Are sufficient details of methods and materials provided to allow replication by others?

Yes

Is the rationale for creating the dataset(s) clearly described?

Yes

Are the datasets clearly presented in a useable and accessible format?

Partly

Are the protocols appropriate and is the work technically sound?

Yes

Reviewer Expertise:

Infectious disease epidemiology

I confirm that I have read this submission and believe that I have an appropriate level of expertise to confirm that it is of an acceptable scientific standard.

# Reviewer report: Reviewer response for version 2

**DOI:** 10.21956/wellcomeopenres.25302.r94001

**Published:** 2024-09-03

**Authors:** Rebecca Cannings-John

**Affiliations:** 1 Cardiff University, Cardiff, Wales, UK

Thank you for answering my queries from last review. I am happy that all have been adequately answered .

Are sufficient details of methods and materials provided to allow replication by others?

Partly

Is the rationale for creating the dataset(s) clearly described?

Yes

Are the datasets clearly presented in a useable and accessible format?

Yes

Are the protocols appropriate and is the work technically sound?

Yes

Reviewer Expertise:

Statistician

I confirm that I have read this submission and believe that I have an appropriate level of expertise to confirm that it is of an acceptable scientific standard.

# Reviewer report: Reviewer response for version 1

**DOI:** 10.21956/wellcomeopenres.22459.r84212

**Published:** 2024-06-11

**Authors:** Rebecca Cannings-John

**Affiliations:** 1 Cardiff University, Cardiff, Wales, UK

This cohort profile paper details the contents of a dynamic open cohort using routinely collected data at care home and individual level with the availability of further analysis of blood samples via the VIVALDI biobank. There are some details that would need confirming/clarification before the article is deemed scientifically sound, especially surrounding the cohort numbers. 

**General comments**

- Where it states “Biobank” or “study Biobank”, could this be renamed as VIVALDI biobank, so not to confuse with the UK Biobank?
- The abstract states that *“This paper aims to describe the samples stored within the study biobank, available for use by researchers.”*  Should this be “samples stored within the study biobank and associated linked data”?

**Consent paragraph: **

- Please provide a link/reference to the UK Biocentre.
- Since the n’s differ for each research question (COVID related and around ageing), I assume that there were two separate questions for the two different levels of consent. Is that correct? 
- It states: *“(n=1,290, 389 residents, 887 staff) or to wider research around ageing (n=974, 438 residents, 528 staff” *- what does the n=1,290 relate to here? Are they 1,290 samples from 389 residents and 887 staff or is the n the total number of participants? If the latter then the numbers don’t add up: 389+887=1276.
- Also, these numbers stated as consented are different to the N of 2,268 staff and residents, and 166 care homes in Figure 1.

**Data linkage cohort **

- Please add the N individuals from 346 care homes or is there a reason why there is no definitive N?
- I can see that detailed linkage information is reported under “What has been measured?” – it would be useful to refer to this section in this paragraph.

**Figure 1**

- Same point as before; ~70,000 – why isn’t the exact number reported? (same for the note under the title of Figure 1 and also ~7,000 staff – please add exact numbers)
- The VIVALDI biobank Ns are 2,268 staff and residents from 166 care homes. However, the note under the title states “2,396 individuals from 167 care homes”.

**Immunological cohort**

- “Linkage to routine data” – again more detail on which data sources/datasets, quality of linkage etc are needed so please refer to the “What has been measured?” paragraph. 

**Stored samples**

- “There were a total of 1,371 participants in whom SARS-CoV-2 infection was recorded” – what is the denominator here? The 2,268? Please add in the %.

**How often have they been followed up?**

- “How often have they been followed up?” – could you change ‘they’ to participants?  

**What has been measured?**

- Could this section be written in the same order as the cohorts are listed (i.e. data linkage, immune, biobank) so it is clear what data is coming from which cohort and at what level. For example the section starting “linkage was also undertaken using CQC-IDs from participating care homes to the Capacity Tracker dataset …” should come under the first paragraph of data linkage (I think??).
- Detail should be provided on the individual data linkage rates for assurances around quality/coverage.

**Table 2**

- Could a note be added to explain what Pillar 1 and 2 mean?

Are sufficient details of methods and materials provided to allow replication by others?

Partly

Is the rationale for creating the dataset(s) clearly described?

Yes

Are the datasets clearly presented in a useable and accessible format?

Yes

Are the protocols appropriate and is the work technically sound?

Yes

Reviewer Expertise:

Trial statistician with expertise in routine data

I confirm that I have read this submission and believe that I have an appropriate level of expertise to confirm that it is of an acceptable scientific standard, however I have significant reservations, as outlined above.

# Response

**Published:** 2024-08-12

**Authors:** Maria Krutikov

**Affiliations:** Institute of Health Informatics, University College London, London, UK

**General comments **Where it states “Biobank” or “study Biobank”, could this be renamed as VIVALDI biobank, so not to confuse with the UK Biobank?

**Response: **Thank you for the suggestion, this has been updated throughout.

**Comment:** The abstract states that “This paper aims to describe the samples stored within the study biobank, available for use by researchers.”  Should this be “samples stored within the study biobank and associated linked data”?

**Response: **Thank you for the suggestion, we have added this.

**Comment: Consent paragraph:**

- Please provide a link/reference to the UK Biocentre.
**Response: ** This has been added.

**Comment:** Since the n’s differ for each research question (COVID related and around ageing), I assume that there were two separate questions for the two different levels of consent. Is that correct? 

**Response: **Yes this is correct. We have updated the text to add “using two different consent questions”

**Comment:** It states: “(n=1,290, 389 residents, 887 staff) or to wider research around ageing (n=974, 438 residents, 528 staff” - what does the n=1,290 relate to here? Are they 1,290 samples from 389 residents and 887 staff or is the n the total number of participants? If the latter then the numbers don’t add up: 389+887=1276.

**Response: **Thank you for highlighting this point. There were 1290 participants who consented to sample storage for COVID-19 research only. When broken down, there were 14 individuals in whom it was not known whether they were staff or residents. This has been added to the text. “All samples belong to participants who have consented to their further use by researchers outside of the VIVALDI team (n=2264, 1415 staff, 827 residents, 22 unknown) using two different consent questions, either for research relating to COVID-19 only (n=1,290, 389 residents, 887 staff, 14 unknown) or to wider research around ageing (n=974, 438 residents, 528 staff, 8 unknown).” When reviewing these figures, we identified an error in the original data cleaning which resulted in the inclusion of 4 staff members who had not consented to longer term sample storage. We have therefore removed these individuals from the Vivaldi biobank cohort and updated the numbers, tables, and figures throughout the text accordingly.

**Comment:** Also, these numbers stated as consented are different to the N of 2,268 staff and residents, and 166 care homes in Figure 1.

**Response: **The numbers in the consent paragraph describe the breakdown of different types of consent amongst the participants with stored samples (COVID-19 research only vs wider research)

**Comment: Data linkage cohort**

 Please add the N individuals from 346 care homes or is there a reason why there is no definitive N?

**Response: **As the VIVALDI cohort used data linkage to routine datasets, the quality of the data was limited by issues with data entry within those national datasets which affected our ability to link everyone from participating care homes to a valid pseudo-identifier. This is because NHS numbers were often not entered or incorrectly entered by the participant when requesting COVID-19 PCRs or entering lateral flow results through the national government testing portal. As such, we were unable to accurately link these tests to an individual so have only been able to estimate the number of participants. However, we have added the total count of individuals who could be linked to a valid NHS number to Figure 1 (n=72,183). We have added the following sentence to the Data Linkage Cohort section: “The precision of estimates of the exact number of participants are limited by data entry errors or incomplete data, however 72,183 participants could be linked to a valid NHS number.”

**Comment: **I can see that detailed linkage information is reported under “What has been measured?” – it would be useful to refer to this section in this paragraph.

**Response: **Thank you, we have added this reference to the text “described in more detail in “what has been measured?"

**Comment: Figure 1**

- Same point as before; ~70,000 – why isn’t the exact number reported? (same for the note under the title of Figure 1 and also ~7,000 staff – please add exact numbers)

**Response: **See response to point under “data linkage cohort” section for the data linkage cohort. We have updated the legend for Figure 1 to show 7038 participants, we thank the reviewer for this useful feedback.

**Comment: **The VIVALDI biobank Ns are 2,268 staff and residents from 166 care homes. However, the note under the title states “2,396 individuals from 167 care homes”.

**Response: **Thank you for highlighting this omission, we have amended the error.

**Comment:  Immunological cohort**

 Linkage to routine data” – again more detail on which data sources/datasets, quality of linkage etc are needed so please refer to the “What has been measured?” paragraph. 

**Response: **Thank you, we have added this reference “detailed description in “what has been measured?” section”

**Comment:**

**Stored samples**

- “There were a total of 1,371 participants in whom SARS-CoV-2 infection was recorded” – what is the denominator here? The 2,268? Please add in the %.

**Response: ** Yes, this has been amended accordingly.

**Comment: How often have they been followed up?**

- “How often have they been followed up?” – could you change ‘they’ to participants?  

**Response: **This has been updated

**Comment: What has been measured?**

- Could this section be written in the same order as the cohorts are listed (i.e. data linkage, immune, biobank) so it is clear what data is coming from which cohort and at what level. For example the section starting “linkage was also undertaken using CQC-IDs from participating care homes to the Capacity Tracker dataset …” should come under the first paragraph of data linkage (I think??).

**Response: ** This section describes the data linkage process for all linked metadata regardless of which cohort the participant belongs to as this process is the same for all of the cohorts. We have amended the section to make this clearer: “Results from immunological testing of blood samples have been linked to the data linkage cohort (pseudonymised study dataset).”

**Comment: **

- Detail should be provided on the individual data linkage rates for assurances around quality/coverage.

**Response: **Thank you for this suggestion. It is challenging to estimate the individual linkage rates as unlinked sampled were only included in the dataset from 1 ^st^ March 2021 onwards (for the final two years of the study). Prior to this, unlinked samples were removed by the data pipelines that formed the final dataset. However from 1 ^st^ March 2021 onwards, 6601/9486 samples could be linked to metadata. We have added the following description to the “what has been measured” section: “From 1 ^st^ March 2021 onwards 69.6% (6601/9486) of all blood samples received could be linked to a valid NHS number. Individual data linkage rates can only be estimated after this date as prior to this unlinked samples were excluded from the dataset. Only linked samples were included in the VIVALDI biobank cohort.”

**Comment:**

**Table 2**

- Could a note be added to explain what Pillar 1 and 2 mean?

**Response: ** We have added a note under table 2: “Pillar 1 – UKHSA-led national COVID-19 testing programme to detect infections during care home outbreaks or during hospital admission. Pillar 2 – DHSC-led national COVID-19 testing programme to detect asymptomatic and symptomatic infections amongst care home staff and residents.”

# Reviewer report: Reviewer response for version 1

**DOI:** 10.21956/wellcomeopenres.22459.r76527

**Published:** 2024-03-28

**Authors:** Sebastian Maurer-Stroh

**Affiliations:** 1 Agency for Science, Technology and Research (A*STAR), Bioinformatics Institute, Matrix, Singapore; 2 National University of Singapore, Singapore, Singapore

This is a cohort profile report of pre- and post-infection samples linked to data on COVID-19 infections, vaccinations, and outcomes of staff and residents in elderly care homes in England during the COVID-19 pandemic.

 Although 70,000 individuals from 346 care homes were included in the data linkage cohort, the core of the cohort with samples collected and available comprises of only 2268 individuals which nevertheless is an important and valuable biobank and dataset.

 Comment 1: 

 Given the opportunistic recruitment and open cohort design, there is risk of sampling biases over time so a proper presentation of the relevant data distributions is important. 

 - For example, Figure 2 appears to suggest oversampling of staff blood samples relative to residents in 2021 but this only appears to be the case because the Y-axis max scale of number of blood samples in 2.c) is different from 2.a) and 2.b). Best to use same max Y in all 3 plots (same for secondary Y-axis number of participants).

 - Since no sampling frames on age or sex were applied, there could be fluctuations over time. Tables 3 and 4 show summary demographics and could be complemented by adding temporal bins or also plotting age and sex over time for residents vs staff. 

 Comment 2:

 The website to express interest for access does not seem to consider options for access to the metadata only. I may be mistaken but it seems that data availability generally is limited to the subset with samples in the biobank. There would be great value for research in providing access to the larger linked data even without samples if permitted.

Are sufficient details of methods and materials provided to allow replication by others?

Yes

Is the rationale for creating the dataset(s) clearly described?

Yes

Are the datasets clearly presented in a useable and accessible format?

Partly

Are the protocols appropriate and is the work technically sound?

Yes

Reviewer Expertise:

Infectious disease epidemiology

I confirm that I have read this submission and believe that I have an appropriate level of expertise to confirm that it is of an acceptable scientific standard, however I have significant reservations, as outlined above.

# Response

**Published:** 2024-08-12

**Authors:** Maria Krutikov

**Affiliations:** Institute of Health Informatics, University College London, London, UK

**Comment 1:**

 Given the opportunistic recruitment and open cohort design, there is risk of sampling biases over time so a proper presentation of the relevant data distributions is important. 

- For example, Figure 2 appears to suggest oversampling of staff blood samples relative to residents in 2021 but this only appears to be the case because the Y-axis max scale of number of blood samples in 2.c) is different from 2.a) and 2.b). Best to use same max Y in all 3 plots (same for secondary Y-axis number of participants).

**Response:**

 Thank you for this feedback. We have updated Figure 2 to show the same primary and secondary Y-axes in all plots. 

**Comment 1: **

- Since no sampling frames on age or sex were applied, there could be fluctuations over time. Tables 3 and 4 show summary demographics and could be complemented by adding temporal bins or also plotting age and sex over time for residents vs staff. 

**Response** ***: *** We thank the reviewer for the suggestion. We have updated Table 3 to include the temporal distribution of sampled and unsampled population. Whilst updating the table we have identified a small error in the cleaning code which resulted in an underestimate of the unsampled population. We have amended the code and updated Table 3 accordingly. The following explanatory comment has been added to the text: “A greater number of samples were obtained from staff than from residents up until April 2022, after which the proportion equalised within the sampled population with slight predominance of staff in the unsampled population over the same period. However smaller numbers were recruited over this period, reflecting a shift in the study’s focus to detailed investigation of immune responses in a sub-cohort from March 2022. As the national testing programme wound down over the final months of the study, the number of COVID-19 tests being conducted dropped rapidly making it more challenging to identify the unsampled care home population within routine datasets.” We have also updated Table 4 to include periods of first sample collection, overall and amongst staff and residents. We have also added the following explanation to the text: “Primary samples were most frequently collected early on before April 2021, least frequently in the final year of the study (1 ^st^ April 2022 – 1 ^st^ April 2023). A larger proportion of participants who donated their first sample before April 2021 provided at least one further sample during the study (1061/1484, 71.5% vs 456/780, 58.5%), although participants entering in the final months of the study may have had fewer opportunities to do so.”    

**Comment 2:**

 The website to express interest for access does not seem to consider options for access to the metadata only. I may be mistaken but it seems that data availability generally is limited to the subset with samples in the biobank. There would be great value for research in providing access to the larger linked data even without samples if permitted.

**Response: **Although the VIVALDI study accessed data from a large care home cohort, we are only able to share metadata linked to stored samples and not for the wider data linkage cohort. This is because of restrictions on data sharing as data for the broader “data linkage cohort” were accessed under the Control of Patient Information Regulations 2002 (COPI) notice. In contrast, participants who donated blood samples gave informed consent for data sharing and sample storage. We have added the following explanatory sentence to data availability section: “In view of restrictions on data sharing, metadata will only be available for linked samples and cannot be shared for the wider data linkage cohort who have not consented to sampling.”
